# Rough ${I}_{2}$-lacunary statistical convergence of double sequences

**DOI:** 10.1186/s13660-018-1831-7

**Published:** 2018-09-10

**Authors:** Ömer Kişi, Erdinç Dündar

**Affiliations:** 1 Department of Mathematics, Faculty of Science, Bartın University, Bartın, Turkey; 2 Department of Mathematics, Faculty of Science, Afyon Kocatepe University, AfyonKarahisar, Turkey

**Keywords:** Statistical convergence, $\mathcal{I}$-convergence, Rough convergence, Lacunary sequences, Double sequences

## Abstract

In this paper, we introduce and study the notion of rough $\mathcal {I}_{2}$-lacunary statistical convergence of double sequences in normed linear spaces. We also introduce the notion of rough $\mathcal{I}_{2}$-lacunary statistical limit set of a double sequence and discuss some properties of this set.

## Introduction

Throughout the paper, $\mathbb{N}$ and $\mathbb{R}$ denote the set of all positive integers and the set of all real numbers, respectively. The concept of convergence of a sequence of real numbers has been extended to statistical convergence independently by Fast [1] and Schoenberg [2]. This concept was extended to the double sequences by Mursaleen and Edely [3]. Lacunary statistical convergence was defined by Fridy and Orhan [4]. Çakan and Altay [5] presented multidimensional analogues of the results presented by Fridy and Orhan [4].

The idea of $\mathcal{I}$-convergence was introduced by Kostyrko et al. [6] as a generalization of statistical convergence which is based on the structure of the ideal $\mathcal{I}$ of subset of the set of natural numbers. Kostyrko et al. [7] studied the idea of $\mathcal{I}$-convergence and extremal $\mathcal{I}$-limit points. Das et al. [8, 9] introduced the concept of $\mathcal{I}$-convergence of double sequences in a metric space and studied some properties of this convergence. A lot of development have been made in area about statistical convergence, $\mathcal{I}$-convergence and double sequences after the work of [1, 2, 10–28].

The notion of lacunary ideal convergence of real sequences was introduced in [29]. Das et al. [30, 31] introduced new notions, namely $\mathcal{I}$-statistical convergence and $\mathcal{I}$-lacunary statistical convergence by using ideal. Belen et al. [32] introduced the notion of ideal statistical convergence of double sequences, which is a new generalization of the notions of statistical convergence and usual convergence. Kumar et al. [33] introduced $\mathcal{I}$-lacunary statistical convergence of double sequences. Further investigation and applications on this notion can be found in [34].

The idea of rough convergence was first introduced by Phu [35] in finite-dimensional normed spaces. In another paper [36] related to this subject, Phu defined the rough continuity of linear operators and showed that every linear operator $f:X\rightarrow Y$ is *r* -continuous at every point $x\in X$ under the assumption $\operatorname{dim}Y<\infty$ and $r>0$, where *X* and *Y* are normed spaces. In [37], Phu extended the results given in [35] to infinite-dimensional normed spaces. Aytar [38] studied the rough statistical convergence. Also, Aytar [39] studied that the rough limit set and the core of a real sequence. Recently, Dündar and Çakan [11, 40], Pal et al. [41] introduced the notion of rough $\mathcal{I}$-convergence and the set of rough $\mathcal{I}$-limit points of a sequence and studied the notion of rough convergence and the set of rough limit points of a double sequence. Further this notion of rough convergence of double sequence has been extended to rough statistical convergence of double sequence by Malik et al. [42] using double natural density of $\mathbb{N\times\mathbb{N}}$ in the similar way as the notion of convergence of double sequence in Pringsheim sense was generalized to statistical convergence of double sequence. Also, Dündar [43] investigated rough $\mathcal{I}_{2}$-convergence of double sequences. The notion of $\mathcal{I}$-statistical convergence of double sequences was introduced by Malik and Ghosh [44] in the theory of rough convergence.

In view of the recent applications of ideals in the theory of convergence of sequences, it seems very natural to extend the interesting concept of rough lacunary statistical convergence further by using ideals which we mainly do here.

So it is quite natural to think, if the new notion of $\mathcal{I}$-lacunary statistical convergence of double sequences can be introduced in the theory of rough convergence.

## Definitions and notations

In this section, we recall some definitions and notations, which form the base for the present study [6, 10, 11, 23, 32, 33, 35, 38, 40, 42–46].

Throughout the paper, let *r* be a nonnegative real number and $\mathbb{R}^{n}$ denotes the real *n*-dimensional space with the norm $\|\cdot\|$. Consider a sequence $x = (x_{i})\subset\mathbb{R}^{n}$.

The sequence $x = (x_{i})$ is said to be *r*-convergent to $x_{*}$, denoted by $x_{i} \overset{r}{\longrightarrow} x_{*}$, provided that

$$ \forall\varepsilon> 0\ \exists i_{\varepsilon}\in\mathbb{N}: i \geq i_{\varepsilon}\quad \Rightarrow \quad \Vert x_{i} - x_{*} \Vert < r+ \varepsilon. $$

The set

$$ \text{LIM}^{r}x:= \bigl\{ x_{\ast}\in\mathbb{R}^{n}:x_{i} \overset{r}{\longrightarrow}x_{\ast} \bigr\} $$

is called the *r*-limit set of the sequence $x=(x_{i})$. A sequence $x=(x_{i})$ is said to be *r*-convergent if $\mathtt{LIM}^{r}x\neq \emptyset$. In this case, *r* is called the convergence degree of the sequence $x=(x_{i})$. For $r=0$, we get the ordinary convergence. There are several reasons for this interest (see [35]).

A family of sets $\mathcal{I}\subseteq2^{\mathbb{N}}$ is called an ideal if and only if

(i) $\emptyset\in\mathcal{I}$,
(ii) for each $A,B\in\mathcal{I}$ we have $A\cup B\in\mathcal{I}$,
(iii) for each $A\in\mathcal{I}$ and each $B\subseteq A$ we have $B\in \mathcal{I}$.

An ideal is called non-trivial if $\mathbb{N}\notin\mathcal{I}$ and a non-trivial ideal is called admissible if $\{n \} \in \mathcal{I} $ for each $n\in\mathbb{N}$.

A family of sets $\mathcal{F}\subseteq2^{\mathbb{N}}$ is a filter in $\mathbb{N}$ if and only if

(i) $\emptyset\notin\mathcal{F}$,
(ii) for each $A,B\in\mathcal{F}$ we have $A\cap B\in\mathcal{F}$,
(iii) for each $A\in\mathcal{F}$ and each $B\supseteq A$ we have $B\in \mathcal{F}$.

If $\mathcal{I}$ is a non-trivial ideal in $\mathbb{N}$ ( i.e., $\mathbb{N}\notin\mathcal{I}$), then the family of sets

$$ \mathcal{F} ( \mathcal{I} ) = \{ M\subset\mathbb {N}:\exists A\in\mathcal{I}:M= \mathbb{N} \setminus A \} $$

is a filter of $\mathbb{N}$ and it is called the filter associated with the ideal $\mathcal{I}$.

A sequence $x = (x_{i})$ is said to be rough $\mathcal{I}$-convergent (*r*-$\mathcal{I}$-convergent) to $x_{*}$ with the roughness degree *r*, denoted by $x_{i} \overset{r\text{-}\mathcal{I}}{\longrightarrow}x_{*}$ provided that $\{i\in \mathbb{N}:\|x_{i} - x_{*}\|\geq r+\varepsilon\}\in\mathcal{I}$ for every $\varepsilon>0$; or equivalently, if the condition

1 $$ \mathcal{I}\text{-}\limsup \Vert x_{i} - x_{*} \Vert \leq r $$

is satisfied. In addition, we can write $x_{i} \overset{r\text{-}\mathcal{I}}{\longrightarrow}x_{*}$ iff the inequality $\|x_{i} - x_{*}\|< r+\varepsilon$ holds for every $\varepsilon>0$ and almost all *i*.

A double sequence $x=(x_{mn})_{(m,n)\in\mathbb{N}\times\mathbb{N}}$ of real numbers is said to be bounded if there exists a positive real number *M* such that $|x_{mn}| < M$, for all $m,n \in\mathbb{N}$. That is

$$ \Vert x \Vert _{\infty}= \sup_{m,n} \vert x_{mn} \vert < \infty. $$

A double sequence $x=(x_{mn})$ of real numbers is said to be convergent to $L \in\mathbb{R}$ in Pringsheim’s sense (shortly, *p*-convergent to $L \in \mathbb{R}$), if for any $\varepsilon>0$, there exists $N_{\varepsilon}\in \mathbb{N}$ such that $|x_{mn} -L| < \varepsilon$, whenever $m,n> N_{\varepsilon}$. In this case, we write

$$ \lim_{m,n \rightarrow\infty} x_{mn} = L. $$

We recall that a subset *K* of $\mathbb{N} \times\mathbb{N}$ is said to have natural density $d(K)$ if

$$ d(K)=\lim_{m,n\to\infty}\frac{K(m,n)}{m.n}, $$

where $K(m,n)=|\{(j,k)\in\mathbb{N} \times\mathbb{N}: j\leq m, k\leq n\}|$.

Throughout the paper we consider a sequence $x = (x_{mn})$ such that $(x_{mn})\in\mathbb{R}^{n}$.

Let $x=(x_{mn})$ be a double sequence in a normed space $(X,\|\cdot\|)$ and *r* be a non-negative real number. *x* is said to be *r*-statistically convergent to *ξ*, denoted by $x\overset{r\text{-st}_{2}}{\longrightarrow}\xi $, if for $\varepsilon> 0$ we have $d(A(\varepsilon))=0$, where $A(\varepsilon)=\{(m,n)\in\mathbb{N} \times\mathbb{N}: \|x_{mn}-\xi\| \geq r+\varepsilon\}$. In this case, *ξ* is called the *r*-statistical limit of *x*.

A non-trivial ideal $\mathcal{I}_{2}$ of $\mathbb{N} \times\mathbb{N}$ is called strongly admissible if $\{i\}\times\mathbb{N}$ and $\mathbb {N}\times \{i\} $ belong to $\mathcal{I}_{2}$ for each $i \in\mathbb{N}$.

It is evident that a strongly admissible ideal is admissible also.

Throughout the paper we take $\mathcal{I}_{2}$ as a strongly admissible ideal in $\mathbb{N} \times\mathbb{N}$.

Let $(X, \rho)$ be a metric space A double sequence $x= (x_{mn})$ in *X* is said to be $\mathcal{I}_{2}$-convergent to $L \in X$, if for any $\varepsilon >0$ we have $A(\varepsilon) = \{(m,n) \in\mathbb{N} \times\mathbb{N} : \rho(x_{mn} , L) \geq\varepsilon\} \in\mathcal{I}_{2}$. In this case, we say that *x* is $\mathcal{I}_{2}$-convergent and we write

$$ \mathcal{I}_{2}\text{-}\lim_{m,n \rightarrow\infty} x_{mn} = L. $$

A double sequence $x = (x_{mn})$ is said to be rough convergent (*r*-convergent) to $x_{*}$ with the roughness degree *r*, denoted by $x_{mn} \overset{r}{\longrightarrow} x_{*}$ provided that

2 $$ \forall\varepsilon> 0\ \exists k_{\varepsilon}\in\mathbb{N}: m,n \geq k_{\varepsilon} \quad\Rightarrow \quad \Vert x_{mn} - x_{*} \Vert < r+ \varepsilon, $$

or equivalently, if

3 $$ \limsup \Vert x_{mn} - x_{*} \Vert \leq r. $$

A double sequence $x = (x_{mn})$ is said to be *r*-$\mathcal{I}_{2}$-convergent to $x_{*}$ with the roughness degree *r*, denoted by $x_{mn} \overset{r\text{-}\mathcal{I}_{2}}{\longrightarrow}x_{*}$ provided that

4 $$ \bigl\{ (m,n) \in\mathbb{N} \times\mathbb{N}: \Vert x_{mn} - x_{*} \Vert \geq r+\varepsilon \bigr\} \in\mathcal{I}_{2}, $$

for every $\varepsilon>0$; or equivalently, if the condition

5 $$ \mathcal{I}_{2}\text{-}\limsup \Vert x_{mn} - x_{*} \Vert \leq r $$

is satisfied. In addition, we can write $x_{mn} \overset{r\text{-}\mathcal {I}_{2}}{\longrightarrow}x_{*}$ iff the inequality $\|x_{mn} - x_{*}\|< r+\varepsilon$ holds for every $\varepsilon>0$ and almost all $(m,n)$.

Now, we give the definition of $\mathcal{I}_{2}$-asymptotic density of $\mathbb{N}\times\mathbb{N}$.

A subset $K\subset\mathbb{N}\times\mathbb{N}$ is said to be have $\mathcal{I}_{2}$-asymptotic density $d_{\mathcal{I}_{2}} (K ) $ if

$$ d_{\mathcal{I}_{2}} ( K ) =\mathcal{I}_{2}\text{-}\lim _{m,n\rightarrow \infty}\frac{ \vert K ( m,n ) \vert }{m.n}, $$

where $K ( m,n ) = \{ ( j,k ) \in\mathbb {N}\times \mathbb{N}:j\leq m,k\leq n; ( j,k ) \in K \} $ and $\vert K (m,n ) \vert $ denotes number of elements of the set $K(m,n) $.

A double sequence $x= \{ x_{jk} \}$ of real numbers is $\mathcal{I}_{2}$-statistically convergent to *ε*, and we write $x\overset{ \mathcal{I}_{2}\text{-st}}{\rightarrow}\xi$, provided that for any $\varepsilon>0$ and $\delta>0$

$$ \biggl\{ ( m,n ) \in\mathbb{N\times\mathbb{N}}\text{:}\frac {1}{mn} \bigl\vert \bigl\{ ( j,k ) : \Vert x_{jk}-\xi \Vert \geq \varepsilon\text{, }j\leq m,k\leq n \bigr\} \bigr\vert \geq\delta \biggr\} \in \mathcal{I}_{2}\text{.} $$

Let $x= \{ x_{jk} \}$ be a double sequence in a normed linear space $( X, \Vert \cdot \Vert ) $ and *r* be a non-negative real number. Then *x* is said to be rough $\mathcal{I}_{2}$-statistical convergent to *ξ* or *r*-$\mathcal{I}_{2}$-statistical convergent to *ξ* if for any $\varepsilon>0$ and $\delta>0$

$$ \biggl\{ ( m,n ) \in\mathbb{N\times\mathbb{N}}\text{:}\frac {1}{mn} \bigl\vert \bigl\{ ( j,k ) \text{, }j\leq m,k\leq n: \Vert x_{jk}- \xi \Vert \geq r+\varepsilon \bigr\} \bigr\vert \geq \delta \biggr\} \in \mathcal{I}_{2}\text{.} $$

In this case, *ξ* is called the rough $\mathcal{I}_{2}$-statistical limit of $x= \{ x_{jk} \}$ and we denote it by $x\overset {r\text{-}\mathcal{I}_{2}\text{-st}}{\longrightarrow}\xi$.

A double sequence $\overline{\theta}=\theta_{us}= \{ ( k_{u},l_{s} ) \} $ is called a double lacunary sequence if there exist two increasing sequences of integers $( k_{u} ) $ and $( l_{s} ) $ such that

$$ k_{0}=0,\qquad h_{u}=k_{u}-k_{u-1} \rightarrow\infty\quad \text{and}\quad l_{0}=0,\qquad \overline{h}_{s}=l_{s}-l_{s-1} \rightarrow\infty, \quad u,s\rightarrow\infty. $$

We will use the notation $k_{us}:=k_{u}l_{s}$, $h_{us}:=h_{u}\overline{h}_{s}$ and $\theta_{us}$ is determined by

$$ \begin{gathered} J_{us}:= \bigl\{ ( k,l ) :k_{u-1}< k\leq k_{u} \text{ and }l_{s-1}< l\leq l_{s} \bigr\} , \\ q_{u}:=\frac{k_{u}}{k_{u-1}},\qquad \overline{q}_{s}:= \frac {l_{s}}{l_{s-1}}\quad\text{and}\quad q_{us}:=q_{u} \overline{q}_{s}\text{.}\end{gathered} $$

Throughout the paper, by $\theta_{2}=\theta_{us}= \{ ( k_{u},l_{s} ) \} $ we will denote a double lacunary sequence of positive real numbers, respectively, unless otherwise stated.

A double sequence $x= \{x_{mn} \} $ of numbers is said to be $\mathcal{I}_{2}$-lacunary statistical convergent or $S_{\theta _{2}} ( \mathcal{I}_{2} )$-convergent to *L*, if for each $\varepsilon>0$ and $\delta>0$,

$$ \biggl\{ ( u,s ) \in\mathbb{N}\times\mathbb{N}:\frac {1}{h_{us}} \bigl\vert \bigl\{ ( m,n ) \in J_{us}: \vert x_{mn}-L \vert \geq\varepsilon \bigr\} \bigr\vert \geq\delta \biggr\} \in \mathcal{I}_{2}\text{.} $$

In this case, we write $x_{mn}\rightarrow L ( S_{\theta_{2}} ( \mathcal{I}_{2} ) ) $ or $S_{\theta_{2}} ( \mathcal{I}_{2} ) $-$\lim_{m,n\rightarrow\infty}x_{mn}=L$.

## Main results

### Definition 3.1

Let $x= \{ x_{jk} \}$ be a double sequence in a normed linear space $( X, \Vert \cdot \Vert ) $ and *r* be a non-negative real number. Then *x* is said to be rough lacunary statistical convergent to *ξ* or *r*-lacunary statistical convergent to *ξ* if for any $\varepsilon>0$

$$ \lim_{u,s\rightarrow\infty}\frac{1}{h_{us}} \bigl\vert \bigl\{ ( j,k ) \in J_{us}: \Vert x_{jk}-\xi \Vert \geq r+\varepsilon \bigr\} \bigr\vert =0\text{.} $$

In this case *ξ* is called the rough lacunary statistical limit of $x= \{ x_{jk} \} $ and we denote it by $x\overset{r\text{-}S_{\theta _{2}}}{\longrightarrow}\xi$.

### Definition 3.2

Let $x= \{ x_{jk} \} $ be a double sequence in a normed linear space $( X, \Vert \cdot \Vert ) $ and *r* be a non-negative real number. Then *x* is said to be rough $\mathcal{I}_{2}$-lacunary statistical convergent to *ξ* or *r*-$\mathcal{I}_{2}$-lacunary statistical convergent to *ξ* if for any $\varepsilon>0$ and $\delta>0$

$$ \biggl\{ ( u,s ) \in\mathbb{N}\times\mathbb{N}:\frac {1}{h_{us}} \bigl\vert \bigl\{ ( j,k ) \in J_{us}: \Vert x_{jk}-\xi \Vert \geq r+\varepsilon \bigr\} \bigr\vert \geq\delta \biggr\} \in \mathcal{I}_{2}\text{.} $$

In this case, *ξ* is called the rough $\mathcal{I}_{2}$-lacunary statistical limit of $x= \{ x_{jk} \} $ and we denote it by $x \overset{r\text{-}\mathcal{I}_{\theta_{2}}\text{-st}}{\longrightarrow}\xi$.

### Remark 3.3

Note that if $\mathcal{I}_{2}$ is the ideal

$$ \mathcal{I}_{2}^{0}= \bigl\{ A\subset\mathbb{N}\times \mathbb{N}:\exists m ( A ) \in\mathbb{N}\text{ such that }i,j\geq m ( A ) \Rightarrow ( i,j ) \notin A \bigr\} \text{,} $$

then rough $\mathcal{I}_{2}$-lacunary statistical convergence coincides with rough lacunary statistical convergence.

Here *r* in the above definition is called the roughness degree of the rough $\mathcal{I}_{2}$-lacunary statistical convergence. If $r=0$, we obtain the notion of $\mathcal{I}_{2}$-lacunary convergence. But our main interest is when $r>0$. It may happen that a double sequence $x= \{ x_{jk} \}$ is not $\mathcal{I}_{2}$-lacunary statistical convergent in the usual sense, but there exists a double sequence $y= \{ y_{jk} \}$, which is $\mathcal{I}_{2}$-lacunary statistically convergent and satisfying the condition $\Vert x_{jk}-y_{jk} \Vert \leq r$ for all $(j,k)$. Then *x* is rough $\mathcal{I}_{2}$-lacunary statistically convergent to the same limit.

From the above definition it is clear that the rough $\mathcal{I}_{2}$-lacunary statistical limit of a double sequence is not unique. So we consider the set of rough $\mathcal{I}_{2}$-lacunary statistical limits of a double sequence *x* and we use the notation $\mathcal{I}_{\theta _{2}}\text{-st-} \operatorname{LIM}_{x}^{r}$ to denote the set of all rough $\mathcal{I}_{2}$-lacunary statistical limits of a double sequence *x*. We say that a double sequence *x* is rough $\mathcal{I}_{2}$-lacunary statistically convergent if $\mathcal{I}_{\theta_{2}}\text{-st-}\operatorname{LIM}_{x}^{r}\neq\emptyset$.

Throughout the paper *X* denotes a normed linear space $( X, \Vert \cdot \Vert ) $ and *x* denotes the double sequence $\{ x_{jk} \}$ in *X*.

Now, we discuss some basic properties of rough $\mathcal{I}_{2}$-lacunary statistically convergence of double sequences.

### Theorem 3.4

*Let* $x= \{ x_{jk} \}$ *be a double sequence and* $r\geq0$. *Then* $\mathcal{I}_{\theta_{2}}\textit{-st-}\operatorname{LIM}_{x}^{r}\leq2r$. *In particular if* *x* *is rough* $\mathcal{I}_{2}$-*lacunary statistically convergent to* *ξ*, *then*

$$ \mathcal{I}_{\theta_{2}}\textit{-st-}\operatorname{LIM}_{x}^{r}= \overline{B_{r} ( \xi ) }, $$

*where* $\overline{B_{r} ( \xi ) } = \{ y\in X: \Vert y-\xi \Vert \leq r \}$ *and so*

$$ \operatorname{diam} \bigl( \mathcal{I}_{\theta_{2}}\textit{-st-} \operatorname{LIM}_{x}^{r} \bigr) =2r. $$

### Proof

Let $\operatorname{diam} ( \mathcal{I}_{\theta_{2}}\text{-st-}\operatorname{LIM}_{x}^{r} ) >2r$. Then there exist $y,z\in\mathcal{I}_{\theta_{2}}\text{-st-}\operatorname{LIM}_{x}^{r}$ such that $\Vert y-z \Vert >2r$. Now, we select $\varepsilon>0$ so that $\varepsilon<\frac{ \Vert y-z \Vert }{2}-r$. Let

$$ A= \bigl\{ ( j,k ) \in J_{us}: \Vert x_{jk}-y \Vert \geq r+\varepsilon \bigr\} $$

and

$$ B= \bigl\{ ( j,k ) \in J_{us}: \Vert x_{jk}-z \Vert \geq r+\varepsilon \bigr\} \text{.} $$

Then

$$ \begin{gathered} \frac{1}{h_{us}} \bigl\vert \bigl\{ ( j,k ) \in J_{us}: ( j,k ) \in A\cup B \bigr\} \bigr\vert \\ \quad\leq\frac{1}{h_{us}} \bigl\vert \bigl\{ ( j,k ) \in J_{us}: ( j,k ) \in A \bigr\} \bigr\vert +\frac{1}{h_{us}} \bigl\vert \bigl\{ ( j,k ) \in J_{us}: ( j,k ) \in B \bigr\} \bigr\vert \text{,}\end{gathered} $$

and so by the property of $\mathcal{I}_{2}$-convergence

$$ \begin{gathered} \mathcal{I}_{2}\text{-}\lim _{u,s\rightarrow\infty}\frac{1}{h_{us}} \bigl\vert \bigl\{ ( j,k ) \in J_{us}: ( j,k ) \in A\cup B \bigr\} \bigr\vert \\ \quad\leq\mathcal{I}_{2}\text{-}\lim_{u,s\rightarrow\infty} \frac{1}{h_{us}} \bigl\vert \bigl\{ ( j,k ) \in J_{us}: ( j,k ) \in A \bigr\} \bigr\vert \\ \qquad{}+\mathcal{I}_{2}\text{-}\lim_{u,s\rightarrow\infty} \frac{1}{h_{us}} \bigl\vert \bigl\{ ( j,k ) \in J_{us}: ( j,k ) \in B \bigr\} \bigr\vert \\ \quad=0\text{.}\end{gathered} $$

Thus,

$$ \biggl\{ ( u,s ) \in\mathbb{N}\times\mathbb{N}:\frac {1}{h_{us}} \bigl\vert \bigl\{ ( j,k ) \in J_{us}: ( j,k ) \in A\cup B \bigr\} \bigr\vert \geq\delta \biggr\} \in\mathcal{I}_{2} $$

for all $\delta>0$. Let

$$ H= \biggl\{ ( u,s ) \in\mathbb{N}\times\mathbb{N}:\frac {1}{h_{us}} \bigl\vert \bigl\{ ( j,k ) \in J_{us}: ( j,k ) \in A\cup B \bigr\} \bigr\vert \geq\frac{1}{2} \biggr\} \text{.} $$

Clearly $H\in\mathcal{I}_{2}$, so choose $( u_{0},s_{0} ) \in \mathbb{N}\times\mathbb{N}\setminus H$. Then

$$ \frac{1}{h_{u_{0}s_{0}}} \bigl\vert \bigl\{ ( j,k ) \in J_{us}: ( j,k ) \in A\cup B \bigr\} \bigr\vert < \frac {1}{2}\text{.} $$

So, we have

$$ \frac{1}{h_{u_{0}s_{0}}} \bigl\vert \bigl\{ ( j,k ) \in J_{us}: ( j,k ) \notin A\cup B \bigr\} \bigr\vert \geq 1-\frac{1}{2}=\frac{1}{2}, $$

i.e., $\{ ( j,k ) \in J_{us}: ( j,k ) \notin A\cup B \} $ is a nonempty set.

Take $( j_{0},k_{0} ) \in J_{us}$ such that $( j_{0},k_{0} ) \notin A\cup B$. Then $( j_{0},k_{0} ) \in$ $A^{c}\cap B^{c}$ and so $\Vert x_{j_{0}k_{0}}-y \Vert < r+\varepsilon$ and $\Vert x_{j_{0}k_{0}}-z \Vert < r+\varepsilon$. Hence, we have

$$ \begin{aligned} \Vert y-z \Vert &\leq \Vert x_{j_{0}k_{0}}-y \Vert + \Vert x_{j_{0}k_{0}}-z \Vert \\ &\leq 2 ( r+\varepsilon ) \\ &\leq \Vert y-z \Vert \text{,}\end{aligned} $$

which is absurd. Therefore, $\mathcal{I}_{\theta _{2}}\text{-st-}\operatorname{LIM}_{x}^{r}\leq 2r$.

If $\mathcal{I}_{\theta_{2}}\text{-st-}\operatorname{LIM}_{x}^{r}=\xi$, then we proceed as follows. Let $\varepsilon>0$ and $\delta>0$ be given. Then

$$ A= \biggl\{ ( u,s ) \in\mathbb{N}\times\mathbb{N}:\frac {1}{h_{us}} \bigl\vert \bigl\{ ( j,k ) \in J_{us}: \Vert x_{jk}-\xi \Vert \geq\varepsilon \bigr\} \bigr\vert \geq\delta \biggr\} \in \mathcal{I}_{2}\text{.} $$

Then for $( u,s ) \notin A$ we have

$$ \frac{1}{h_{us}} \bigl\vert \bigl\{ ( j,k ) \in J_{us}: \Vert x_{jk}-\xi \Vert \geq\varepsilon \bigr\} \bigr\vert < \delta \text{,} $$

i.e.,

6 $$ \frac{1}{h_{us}} \bigl\vert \bigl\{ ( j,k ) \in J_{us}: \Vert x_{jk}-\xi \Vert < \varepsilon \bigr\} \bigr\vert \geq1-\delta. $$

Now, for each $y\in\overline{B_{r} ( \xi ) }$ we have

7 $$ \Vert x_{jk}-y \Vert \leq \Vert x_{jk}-\xi \Vert + \Vert \xi-y \Vert \leq \Vert x_{jk}-\xi \Vert +r \text{.} $$

Let

$$ B_{us}= \bigl\{ ( j,k ) \in J_{us}: \Vert x_{jk}-\xi \Vert < \varepsilon \bigr\} \text{.} $$

Then for $( j,k ) \in B_{us}$ we have $\Vert x_{jk}-y \Vert < r+\varepsilon$. Hence, we have

$$ B_{us}= \bigl\{ ( j,k ) \in J_{us}: \Vert x_{jk}-y \Vert < r+\varepsilon \bigr\} \text{.} $$

This implies

$$ \frac{ \vert B_{us} \vert }{h_{us}}\leq\frac{1}{h_{us}} \bigl\vert \bigl\{ ( j,k ) \in J_{us}: \Vert x_{jk}-y \Vert < r+\varepsilon \bigr\} \bigr\vert , $$

i.e.,

$$ \frac{1}{h_{us}} \bigl\vert \bigl\{ ( j,k ) \in J_{us}: \Vert x_{jk}-y \Vert < r+\varepsilon \bigr\} \bigr\vert \geq1-\delta \text{.} $$

Thus, for all $( j,k ) \notin A$,

$$ \frac{1}{h_{us}} \bigl\vert \bigl\{ ( j,k ) \in J_{us}: \Vert x_{jk}-y \Vert \geq r+\varepsilon \bigr\} \bigr\vert < 1- ( 1-\delta ) $$

and so we have

$$ \biggl\{ ( u,s ) \in\mathbb{N}\times\mathbb{N}:\frac {1}{h_{us}} \bigl\vert \bigl\{ ( j,k ) \in J_{us}; \Vert x_{jk}-y \Vert \geq r+\varepsilon \bigr\} \bigr\vert \geq\delta \biggr\} \subset A \text{.} $$

Since $A\in\mathcal{I}_{2}$

$$ \biggl\{ ( u,s ) \in\mathbb{N}\times\mathbb{N}:\frac {1}{h_{us}} \bigl\vert \bigl\{ ( j,k ) \in J_{us}: \Vert x_{jk}-y \Vert \geq r+\varepsilon \bigr\} \bigr\vert \geq\delta \biggr\} \in \mathcal{I}_{2}\text{.} $$

This shows that $y\in\mathcal{I}_{\theta_{2}}\text{-st-}\operatorname{LIM}_{x}^{r}$. Therefore, $\mathcal{I}_{\theta_{2}}\text{-st-}\operatorname{LIM}_{x}^{r}\supset \overline{B_{r} ( \xi ) }$.

Conversely, let $y\in\mathcal{I}_{\theta_{2}}\text{-st-}\operatorname{LIM}_{x}^{r}$, $\Vert y-\xi \Vert >r$ and $\varepsilon=\frac{ \Vert y-\xi \Vert -r}{2}$. Now, we take

$$ M_{1}= \bigl\{ ( j,k ) \in J_{us}: \Vert x_{jk}-y \Vert \geq r+\varepsilon \bigr\} $$

and

$$ M_{2}= \bigl\{ ( j,k ) \in J_{us}: \Vert x_{jk}-\xi \Vert \geq\varepsilon \bigr\} \text{.} $$

Then

$$ \begin{gathered} \frac{1}{h_{us}} \bigl\vert \bigl\{ ( j,k ) \in J_{us}: ( j,k ) \in M_{1}\cup M_{2} \bigr\} \bigr\vert \\ \quad\leq\frac{1}{h_{us}} \bigl\vert \bigl\{ ( j,k ) \in J_{us}: ( j,k ) \in M_{1} \bigr\} \bigr\vert +\frac{1}{h_{us}} \bigl\vert \bigl\{ ( j,k ) \in J_{us}: ( j,k ) \in M_{2} \bigr\} \bigr\vert \text{,}\end{gathered} $$

and by the property of $\mathcal{I}_{2}$-convergence

$$\begin{aligned}& \mathcal{I}_{2}\text{-}\lim_{u,s\rightarrow\infty} \frac{1}{h_{us}} \bigl\vert \bigl\{ ( j,k ) \in J_{us}: ( j,k ) \in M_{1} \cup M_{2} \bigr\} \bigr\vert \\& \quad =\mathcal{I}_{2}\text{-} \lim_{u,s\rightarrow\infty} \frac{1}{h_{us}} \bigl\vert \bigl\{ ( j,k ) \in J_{us}: ( j,k ) \in M_{1} \bigr\} \bigr\vert \\& \qquad{} +\mathcal{I}_{2}\text{-}\lim_{u,s\rightarrow\infty} \frac{1}{h_{us}} \bigl\vert \bigl\{ ( j,k ) \in J_{us}: ( j,k ) \in M_{2} \bigr\} \bigr\vert \\& \quad=0. \end{aligned}$$

Now, we let

$$ M= \biggl\{ ( u,s ) \in\mathbb{N}\times\mathbb{N}:\frac {1}{h_{us}} \bigl\vert \bigl\{ ( j,k ) : ( j,k ) \in M_{1}\cup M_{2} \bigr\} \bigr\vert \geq\frac{1}{2} \biggr\} \text{.} $$

Clearly $M\in\mathcal{I}_{2}$ and we choose $( u_{0},s_{0} ) \in \mathbb{N}\times\mathbb{N}\setminus M$. Then we have

$$ \frac{1}{h_{u_{0}s_{0}}} \bigl\vert \bigl\{ ( j,k ) : ( j,k ) \in M_{1} \cup M_{2} \bigr\} \bigr\vert < \frac{1}{2}, $$

and so

$$ \frac{1}{h_{u_{0}s_{0}}} \bigl\vert \bigl\{ ( j,k ) : ( j,k ) \notin M_{1}\cup M_{2} \bigr\} \bigr\vert \geq1- \frac {1}{2}=\frac{1}{2}, $$

i.e., $\{ ( j,k ) : ( j,k ) \notin M_{1}\cup M_{2} \} $ is a nonempty set. Let $( j_{0},k_{0} ) \in J_{us}$ such that $( j_{0},k_{0} ) \notin M_{1}\cup M_{2}$. Then $( j_{0},k_{0} ) \in M_{1}^{c}\cap M_{2}^{c}$ and hence $\Vert x_{j_{0}k_{0}}-y \Vert < r+\varepsilon$ and $\Vert x_{j_{0}k_{0}}-\xi \Vert <\varepsilon$. So

$$ \Vert y-\xi \Vert \leq \Vert x_{j_{0}k_{0}}-y \Vert + \Vert x_{j_{0}k_{0}}-\xi \Vert \leq r+2\varepsilon\leq \Vert y-\xi \Vert , $$

which is absurd. Therefore, $\Vert y-\xi \Vert \leq r$ and so $y\in\overline{B_{r} ( \xi ) }$. Consequently, we have

$$ \mathcal{I}_{\theta_{2}}\text{-st-}\operatorname{LIM}_{x}^{r}= \overline{B_{r} ( \xi ) }. $$

 □

### Theorem 3.5

*Let* $x= \{ x_{jk} \} $ *be a double sequence and* $r\geq 0$ *be a real number*. *Then the rough* $\mathcal{I}_{2}$-*lacunary statistical limit set of the double sequence* *x*, *i*.*e*., *the set* $\mathcal {I}_{\theta _{2}}\textit{-st-}\operatorname{LIM}_{x}^{r}$ *is closed*.

### Proof

If $\mathcal{I}_{\theta_{2}}\text{-st-}\operatorname{LIM}_{x}^{r}=\emptyset$, then there is nothing to prove.

Let us assume that $\mathcal{I}_{\theta_{2}}\text{-st-}\operatorname{LIM}_{x}^{r}\neq \emptyset$. Now, consider a double sequence $\{ y_{jk} \} $ in $\mathcal{I}_{\theta_{2}}\text{-st-}\operatorname{LIM}_{x}^{r}$ with $\lim_{j,k\rightarrow\infty}y_{jk}=y$. Choose $\varepsilon>0$ and $\delta>0$. Then there exists $i_{\frac{\varepsilon}{2}}\in\mathbb{N}$ such that for all $j,k\geq i_{\frac{\varepsilon}{2}}$

$$ \Vert y_{jk}-y \Vert < \frac{\varepsilon}{2}. $$

Let $j_{0},k_{0}>i_{\frac{\varepsilon}{2}}$. Then $y_{j_{0}k_{0}}\in \mathcal{I}_{\theta_{2}}\text{-st-}\operatorname{LIM}_{x}^{r}$. Consequently, we have

$$ A= \biggl\{ ( u,s ) \in\mathbb{N}\times\mathbb{N}:\frac {1}{h_{us}} \biggl\vert \biggl\{ ( j,k ) \in J_{us}; \Vert x_{jk}-y_{j_{0}k_{0}} \Vert \geq r+\frac{\varepsilon}{2} \biggr\} \biggr\vert \geq\delta \biggr\} \in \mathcal{I}_{2}. $$

Clearly $M=\mathbb{N}\times\mathbb{N}\setminus A$ is nonempty, choose $( u,s ) \in M$. We have

$$ \frac{1}{h_{us}} \biggl\vert \biggl\{ ( j,k ) \in J_{us}: \Vert x_{jk}-y_{j_{0}k_{0}} \Vert \geq r+\frac{\varepsilon}{2} \biggr\} \biggr\vert < \delta $$

and so

$$ \frac{1}{h_{us}} \biggl\vert \biggl\{ ( j,k ) \in J_{us}: \Vert x_{jk}-y_{j_{0}k_{0}} \Vert < r+\frac{\varepsilon}{2} \biggr\} \biggr\vert \geq1-\delta\text{.} $$

Put

$$ B_{us}= \biggl\{ ( j,k ) \in J_{us}: \Vert x_{jk}-y_{j_{0}k_{0}} \Vert < r+\frac{\varepsilon}{2} \biggr\} $$

and select $( j,k ) \in B_{us}$. Then we have

$$ \begin{aligned} \Vert x_{jk}-y \Vert &\leq \Vert x_{jk}-y_{j_{0}k_{0}} \Vert + \Vert y_{j_{0}k_{0}}-y \Vert \\ &< r+\frac{\varepsilon}{2}+\frac{\varepsilon}{2} \\ &=r+\varepsilon\text{,}\end{aligned} $$

and so

$$ B_{us}\subset \bigl\{ ( j,k ) \in J_{us}: \Vert x_{jk}-y \Vert < r+\varepsilon \bigr\} \text{,} $$

which implies

$$ 1-\delta\leq\frac{ \vert B_{us} \vert }{h_{us}}\leq\frac {1}{h_{us}} \bigl\vert \bigl\{ ( j,k ) \in J_{us}: \Vert x_{jk}-y \Vert < r+\varepsilon \bigr\} \bigr\vert \text{.} $$

Therefore,

$$ \frac{1}{h_{us}} \bigl\vert \bigl\{ ( j,k ) \in J_{us}: \Vert x_{jk}-y \Vert \geq r+\varepsilon \bigr\} \bigr\vert < 1- ( 1-\delta ) =\delta $$

and so we have

$$ \biggl\{ ( u,s ) \in\mathbb{N}\times\mathbb{N}:\frac {1}{h_{us}} \bigl\vert \bigl\{ ( j,k ) \in J_{us}: \Vert x_{jk}-y \Vert \geq r+\varepsilon \bigr\} \bigr\vert \geq\delta \biggr\} \subset A\in \mathcal{I}_{2}. $$

This shows that $y\in\mathcal{I}_{\theta_{2}}\text{-st-}\operatorname{LIM}_{x}^{r}$. Hence, $\mathcal{I}_{\theta_{2}}\text{-st-}\operatorname{LIM}_{x}^{r}$ is a closed set. □

### Theorem 3.6

*Let* $x= \{ x_{jk} \}$ *be a double sequence and* $r\geq0$ *be a real number*. *Then the rough* $\mathcal{I}_{2}$-*lacunary statistical limit set* $\mathcal{I}_{\theta_{2}}\textit{-st-}LIM_{x}^{r}$ *of the double sequence* *x* *is a convex set*.

### Proof

Let $y_{0},y_{1}\in\mathcal{I}_{\theta_{2}}\text{-st-}\operatorname{LIM}_{x}^{r}$ and $\varepsilon>0$ be given. Let

$$ A_{0}= \bigl\{ ( j,k ) \in J_{us}: \Vert x_{jk}-y_{0} \Vert \geq r+\varepsilon \bigr\} $$

and

$$ A_{1}= \bigl\{ ( j,k ) \in J_{us}: \Vert x_{jk}-y_{1} \Vert \geq r+\varepsilon \bigr\} \text{.} $$

Then by Theorem 3.4, for $\delta>0$ we have

$$ \biggl\{ ( u,s ) \in\mathbb{N}\times\mathbb{N}:\frac {1}{h_{us}} \bigl\vert \bigl\{ ( j,k ) \in J_{us}: (j,k ) \in A_{0}\cup A_{1} \bigr\} \bigr\vert \geq\delta \biggr\} \in\mathcal {I}_{2}\text{.} $$

Now, we choose $0<\delta_{1}<1$ such that $0<1-\delta_{1}<\delta$ and let

$$ A= \biggl\{ ( u,s ) \in\mathbb{N}\times\mathbb{N}:\frac {1}{h_{us}} \bigl\vert \bigl\{ ( j,k ) \in J_{us}: (j,k ) \in A_{0}\cup A_{1} \bigr\} \bigr\vert \geq1-\delta_{1} \biggr\} \text{.} $$

Then $A\in\mathcal{I}_{2}$. For all $( u,s ) \notin A$, we have

$$ \frac{1}{h_{us}} \bigl\vert \bigl\{ ( j,k ) \in J_{us}: ( j,k ) \in A_{0}\cup A_{1} \bigr\} \bigr\vert < 1- \delta_{1} $$

and so

$$ \frac{1}{h_{us}} \bigl\vert \bigl\{ ( j,k ) \in J_{us}: ( j,k ) \notin A_{0}\cup A_{1} \bigr\} \bigr\vert \geq \bigl\{ 1- ( 1-\delta_{1} ) \bigr\} =\delta_{1}\text{.} $$

Therefore, $\{ ( j,k ) : ( j,k ) \notin A_{0}\cup A_{1} \} $ is a nonempty set. Let us take $( j_{0},k_{0} ) \in A_{0}^{c}\cap A_{1}^{c}$ and $0\leq\mu\leq1$. Then

$$ \begin{aligned} \bigl\Vert x_{j_{0}k_{0}}- \bigl[ ( 1-\mu ) y_{0}+\mu y_{1} \bigr] \bigr\Vert & = \bigl\Vert ( 1-\mu ) x_{j_{0}k_{0}}+\mu x_{j_{0}k_{0}}- \bigl[ ( 1-\mu ) y_{0}+\mu y_{1} \bigr] \bigr\Vert \\ & \leq ( 1-\mu ) \Vert x_{j_{0}k_{0}}-y_{0} \Vert +\mu \Vert x_{j_{0}k_{0}}-y_{1} \Vert \\ & < ( 1-\mu ) ( r+\varepsilon ) +\mu ( r+\varepsilon ) =r+\varepsilon \text{.}\end{aligned} $$

Let

$$ M= \bigl\{ ( j,k ) \in J_{us}: \bigl\Vert x_{jk}- \bigl[ ( 1-\mu ) y_{0}+\mu y_{1} \bigr] \bigr\Vert \geq r+ \varepsilon \bigr\} . $$

Then clearly, $A_{0}^{c}\cap A_{1}^{c}\subset M^{c}$. So for $(u,s ) \notin A$, we have

$$ \delta_{1}\leq\frac{1}{h_{us}} \bigl\vert \bigl\{ ( j,k ) \in J_{us}: ( j,k ) \notin A_{0}\cup A_{1} \bigr\} \bigr\vert \leq \frac{1}{h_{us}} \bigl\vert \bigl\{ ( j,k ) \in J_{us}: ( j,k ) \notin M \bigr\} \bigr\vert $$

and so

$$ \frac{1}{h_{us}} \bigl\vert \bigl\{ ( j,k ) \in J_{us}: ( j,k ) \in M \bigr\} \bigr\vert < 1-\delta_{1}< \delta. $$

Therefore,

$$ A^{c}\subset \biggl\{ ( u,s ) \in\mathbb{N}\times\mathbb {N}: \frac{1}{h_{us}} \bigl\vert \bigl\{ ( j,k ) \in J_{us}: (j,k ) \in M \bigr\} \bigr\vert < \delta \biggr\} . $$

Since $A^{c}\in\mathcal{F} ( \mathcal{I}_{2} ) $, we have

$$ \biggl\{ ( u,s ) \in\mathbb{N}\times\mathbb{N}:\frac {1}{h_{us}} \bigl\vert \bigl\{ ( j,k ) \in J_{us}: (j,k ) \in M \bigr\} \bigr\vert < \delta \biggr\} \in\mathcal{F} ( \mathcal{I}_{2} ) $$

and so

$$ \biggl\{ ( u,s ) \in\mathbb{N}\times\mathbb{N}:\frac {1}{h_{us}} \bigl\vert \bigl\{ ( j,k ) : ( j,k )\in M \bigr\} \bigr\vert \geq\delta \biggr\} \in\mathcal{I}_{2}. $$

This completes the proof. □

### Theorem 3.7

*A double sequence* $x= \{ x_{jk} \} $ *is rough* $\mathcal {I}_{2}$-*lacunary statistical convergent to* *ξ* *if and only if there exists a double sequence* $y= \{ y_{jk} \} $ *such that* $\mathcal{I}_{\theta_{2}}\textit{-st-}y=\xi$ *and* $\Vert x_{jk}-y_{jk} \Vert \leq r$, *for all* $( j,k ) \in\mathbb{N}\times\mathbb{N}$.

### Proof

Let $y= \{ y_{jk} \} $ be a double sequence in *X*, which is $\mathcal{I}_{2}$-lacunary statistically convergent to *ξ* and $\Vert x_{jk}-y_{jk} \Vert \leq r$, for all $( j,k ) \in \mathbb{N}\times\mathbb{N}$. Then for any $\varepsilon>0$ and $\delta>0$

$$ A= \biggl\{ ( u,s ) \in\mathbb{N}\times\mathbb{N}:\frac {1}{h_{us}} \bigl\vert \bigl\{ ( j,k ) \in J_{us}: \Vert y_{jk}-\xi \Vert \geq\varepsilon \bigr\} \bigr\vert \geq\delta \biggr\} \in \mathcal{I}_{2}\text{.} $$

Let $( u,s ) \notin A$. Then we have

$$ \frac{1}{h_{us}} \bigl\vert \bigl\{ ( j,k ) \in J_{us}: \Vert y_{jk}-\xi \Vert \geq\varepsilon \bigr\} \bigr\vert < \delta \quad \Rightarrow\quad\frac{1}{h_{us}} \bigl\vert \bigl\{ ( j,k ) \in J_{us}: \Vert y_{jk}-\xi \Vert < \varepsilon \bigr\} \bigr\vert \geq1- \delta\text{.} $$

Now, we let

$$ B_{us}= \bigl\{ ( j,k ) \in J_{us}: \Vert y_{jk}-\xi \Vert < \varepsilon \bigr\} . $$

Then, for $( j,k ) \in B_{us}$, we have

$$ \Vert x_{jk}-\xi \Vert \leq \Vert x_{jk}-y_{jk} \Vert + \Vert y_{jk}-\xi \Vert < r+\varepsilon\text{,} $$

and so

$$ \begin{gathered} B_{us}\subset \bigl\{ ( j,k ) \in J_{us}: \Vert x_{jk}-\xi \Vert < r+\varepsilon \bigr\} \\ \quad\Rightarrow\quad\frac{ \vert B_{us} \vert }{h_{us}}\leq\frac {1}{h_{us}} \bigl\vert \bigl\{ ( j,k ) \in J_{us}: \Vert x_{jk}-\xi \Vert < r+ \varepsilon \bigr\} \bigr\vert \\ \quad\Rightarrow\quad\frac{1}{h_{us}} \bigl\vert \bigl\{ ( j,k ) \in J_{us}: \Vert x_{jk}-\xi \Vert < r+\varepsilon \bigr\} \bigr\vert \geq1-\delta \\ \quad\Rightarrow\quad\frac{1}{h_{us}} \bigl\vert \bigl\{ ( j,k ) \in J_{us}: \Vert x_{jk}-\xi \Vert \geq r+\varepsilon \bigr\} \bigr\vert < 1- ( 1-\delta ) =\delta\text{.}\end{gathered} $$

Thus, we have

$$ \biggl\{ ( u,s ) \in\mathbb{N}\times\mathbb{N}:\frac {1}{h_{us}} \bigl\vert \bigl\{ ( j,k ) \in J_{us}: \Vert x_{jk}-\xi \Vert \geq r+\varepsilon \bigr\} \bigr\vert \geq\delta \biggr\} \subset A $$

and, since $A\in\mathcal{I}_{2}$,

$$ \biggl\{ ( u,s ) \in\mathbb{N}\times\mathbb{N}:\frac {1}{h_{us}} \bigl\vert \bigl\{ ( j,k ) \in J_{us}: \Vert x_{jk}-\xi \Vert \geq r+\varepsilon \bigr\} \bigr\vert \geq\delta \biggr\} \in \mathcal{I}_{2}\text{.} $$

Hence, $\mathcal{I}_{\theta_{2}}\text{-st-}y=\xi$.

Conversely, suppose that $\mathcal{I}_{\theta_{2}}\text{-st-}y=\xi$. Then, for $\varepsilon>0$ and $\delta>0$,

$$ A= \biggl\{ ( u,s ) \in\mathbb{N}\times\mathbb{N}:\frac {1}{h_{us}} \bigl\vert \bigl\{ ( j,k ) \in J_{us}: \Vert x_{jk}-\xi \Vert \geq r+\varepsilon \bigr\} \bigr\vert \geq\delta \biggr\} \in \mathcal{I}_{2}\text{.} $$

Let $( u,s ) \notin A$. Then we have

$$ \frac{1}{h_{us}} \bigl\vert \bigl\{ ( j,k ) \in J_{us}: \Vert x_{jk}-\xi \Vert \geq r+\varepsilon \bigr\} \bigr\vert < \delta $$

and so

$$ \frac{1}{h_{us}} \bigl\vert \bigl\{ ( j,k ) \in J_{us}: \Vert x_{jk}-\xi \Vert < r+\varepsilon \bigr\} \bigr\vert \geq 1-\delta \text{.} $$

Let

$$ B_{us}= \bigl\{ ( j,k ) \in J_{us}: \Vert x_{jk}-\xi \Vert < r+\varepsilon \bigr\} . $$

Now, we define a double sequence $y= \{ y_{jk} \} $ as follows:

$$ y_{jk}=\left \{\textstyle\begin{array}{l@{\quad}l} \xi, & \text{if } \Vert x_{jk}-\xi \Vert \leq r, \\ x_{jk}+r\frac{\xi-x_{jk}}{ \Vert x_{jk}-\xi \Vert } , & \text{otherwise.}\end{array}\displaystyle \right . $$

Then

$$ \textstyle\begin{array}{l@{\quad}l} \Vert y_{jk}-\xi \Vert & =\left \{ \textstyle\begin{array}{l@{\quad}l} 0\text{,} & \text{if } \Vert x_{jk}-\xi \Vert \leq r, \\ \Vert x_{jk}-\xi+r\frac{\xi-x_{jk}}{ \Vert x_{jk}-\xi \Vert } \Vert \text{,} & \text{otherwise,}\end{array}\displaystyle \right . \\ & =\left \{ \textstyle\begin{array}{l@{\quad}l} 0\text{,} & \text{if } \Vert x_{jk}-\xi \Vert \leq r, \\ \Vert x_{jk}-\xi \Vert -r\text{,} & \text{otherwise.}\end{array}\displaystyle \right .\end{array} $$

Let $( j,k ) \in B_{us}$. Then we have

$$ \Vert y_{jk}-\xi \Vert =0,\quad \text{if } \Vert x_{jk}-\xi \Vert \leq r \quad\text{and}\quad \Vert y_{jk}-\xi \Vert < \varepsilon , \quad\text{if }r< \Vert x_{jk}-\xi \Vert < r+\varepsilon $$

and so

$$ B_{us}\subset \bigl\{ ( j,k ) \in J_{us}: \Vert y_{jk}-\xi \Vert < \varepsilon \bigr\} . $$

This implies

$$ \frac{ \vert B_{us} \vert }{h_{us}}\leq\frac {1}{h_{us}} \bigl\vert \bigl\{ ( j,k ) \in J_{us}: \Vert y_{jk}-\xi \Vert < \varepsilon \bigr\} \bigr\vert . $$

Hence, we have

$$ \begin{gathered} \frac{1}{h_{us}} \bigl\vert \bigl\{ ( j,k ) \in J_{us}: \Vert y_{jk}-\xi \Vert < \varepsilon \bigr\} \bigr\vert \geq 1-\delta \\ \quad\Rightarrow\quad\frac{1}{h_{us}} \bigl\vert \bigl\{ ( j,k ) \in J_{us}: \Vert y_{jk}-\xi \Vert \geq\varepsilon \bigr\} \bigr\vert < 1- ( 1-\delta ) =\delta,\end{gathered} $$

and so

$$ \biggl\{ ( u,s ) \in \mathbb{N} \times \mathbb{N} :\frac{1}{h_{us}} \bigl\vert \bigl\{ ( j,k ) \in J_{us}: \Vert x_{jk}-\xi \Vert \geq \varepsilon \bigr\} \bigr\vert \geq \delta \biggr\} \subset A\text{.} $$

Since $A\in\mathcal{I}_{2}$ we have

$$ \biggl\{ ( u,s ) \in\mathbb{N}\times\mathbb{N}:\frac {1}{h_{us}} \bigl\vert \bigl\{ ( j,k ) \in J_{us}: \Vert x_{jk}-\xi \Vert \geq\varepsilon \bigr\} \bigr\vert \geq\delta \biggr\} \in \mathcal{I}_{2}\text{.} $$

So, $\mathcal{I}_{\theta_{2}}\text{-st-}y=\xi$. □

### Definition 3.8

A double sequence $x= \{ x_{jk} \}$ is said to be $\mathcal {I}_{\theta_{2}}$-statistically bounded if there exists a positive number *K* such that for any $\delta>0$ the set

$$ A= \biggl\{ ( u,s ) \in\mathbb{N}\times\mathbb{N}:\frac {1}{h_{us}} \bigl\vert \bigl\{ ( j,k ) \in J_{us}: \Vert x_{jk} \Vert \geq K \bigr\} \bigr\vert \geq\delta \biggr\} \in\mathcal {I}_{2}\text{.} $$

The next result provides a relationship between boundedness and rough $\mathcal{I}_{\theta_{2}}$-statistical convergence of double sequences.

### Theorem 3.9

*If a double sequence* $x= \{ x_{jk} \} $ *is bounded then there exists* $r\geq0$ *such that* $\mathcal{I}_{\theta _{2}}\textit{-st-}\operatorname{LIM}_{x}^{r}\neq \emptyset$.

### Proof

Let $x= \{ x_{jk} \}$ be bounded double sequence. There exists a positive real number *K* such that $\Vert x_{jk} \Vert < K$, for all $( j,k ) \in J_{us}$. Let $\varepsilon>0$ be given. Then

$$ \bigl\{ ( j,k ) \in J_{us}: \Vert x_{jk}-0 \Vert \geq K+ \varepsilon \bigr\} =\emptyset\text{.} $$

Therefore, $0\in\mathcal{I}_{\theta_{2}}\text{-st-}\operatorname{LIM}_{x}^{K}$ and so $\mathcal{I}_{\theta_{2}}\text{-st-}\operatorname{LIM}_{x}^{K}\neq\emptyset$. □

### Remark 3.10

The converse of the above theorem is not true. For example, let us consider the double sequence $x= \{ x_{jk} \} $ in $\mathbb{R} $ defined by

$$ x_{jk=}\left \{ \textstyle\begin{array}{l@{\quad}l} jk, & \text{if }j\text{ and }k\text{ are squares,} \\ 5, & \text{otherwise.}\end{array}\displaystyle \right . $$

Then $\mathcal{I}_{\theta_{2}}\text{-st-}\operatorname{LIM}_{x}^{0}= \{ 5 \} \neq \emptyset$ but the double sequence *x* is unbounded.

### Definition 3.11

A point $\lambda\in X$ is said to be an $\mathcal{I}_{2}$-lacunary statistical cluster point of a double sequence $x= \{ x_{jk} \}$ in *X* if for any $\varepsilon>0$

$$ d_{\mathcal{I}_{2}} \bigl( \bigl\{ ( j,k )\in J_{us} : \Vert x_{jk}-\lambda \Vert < \varepsilon \bigr\} \bigr) \neq0, $$

where

$$ d_{\mathcal{I}_{2}} ( A ) =\mathcal{I}_{2}\text{-}\lim _{u,s\rightarrow \infty}\frac{1}{h_{us}} \bigl\vert \bigl\{ ( j,k ) \in J_{us}: ( j,k ) \in A \bigr\} \bigr\vert , $$

if it exists. The set of $\mathcal{I}_{2}$-lacunary statistical cluster points of *x* is denoted by $\Lambda_{x}^{S_{\theta_{2}}} ( \mathcal {I}_{2} ) $.

### Theorem 3.12

*For any arbitrary* $\alpha\in\Lambda_{x}^{S_{\theta_{2} }} ( \mathcal{I}_{2} ) $ *of a double sequence* $x= \{ x_{jk} \}$ *we have* $\Vert \xi-\alpha \Vert \leq r$, *for all* $\xi\in\mathcal{I}_{\theta_{2}}\textit{-st-}\operatorname{LIM}_{x}^{r}$.

### Proof

Assume that there exists a point $\alpha\in\Lambda_{x}^{S_{\theta _{2}}} ( \mathcal{I}_{2} ) $ and $\xi\in\mathcal{I}_{\theta _{2}}\text{-st-}\operatorname{LIM}_{x}^{r}$ such that $\Vert \xi-\alpha \Vert >r$. Let $\varepsilon=\frac{ \Vert \xi-\alpha \Vert -r}{3}$. Then

8 $$ \bigl\{ ( j,k ) \in J_{us}: \Vert x_{jk}-\xi \Vert \geq r+\varepsilon \bigr\} \supset \bigl\{ ( j,k ) \in J_{us} : \Vert x_{jk}-\alpha \Vert < \varepsilon \bigr\} \text{.} $$

Since $\alpha\in\Lambda_{x}^{S_{\theta_{2}}} ( \mathcal{I}_{2} ) $ we have

$$ d_{\mathcal{I}_{2}} \bigl( \bigl\{ ( j,k )\in J_{us} : \Vert x_{jk}-\alpha \Vert < \varepsilon \bigr\} \bigr) \neq0. $$

Hence by (8) we have

$$ d_{\mathcal{I}_{2}} \bigl( \bigl\{ ( j,k )\in J_{us}: \Vert x_{jk}-\alpha \Vert \geq r+\varepsilon \bigr\} \bigr)\neq0, $$

which contradicts that $\xi\in\mathcal{I}_{\theta_{2}}\text{-st-}\operatorname{LIM}_{x}^{r}$. Hence, $\Vert \xi-\alpha \Vert \leq r$. □

## Conclusion

The rough convergence has recently been studied by several authors. In view of the recent applications of ideals in the theory of convergence of sequences, it seems very natural to extend the interesting concept of rough lacunary statistical convergence further by using ideals, which we mainly do here; and we investigate some properties of this new type of convergence. So, we have extended some well-known results.
